# Metabolic syndrome and myocardium steatosis in subclinical type 2 diabetes mellitus: a ^1^H-magnetic resonance spectroscopy study

**DOI:** 10.1186/s12933-020-01044-1

**Published:** 2020-05-29

**Authors:** Yue Gao, Yan Ren, Ying-kun Guo, Xi Liu, Lin-jun Xie, Li Jiang, Meng-ting Shen, Ming-yan Deng, Zhi-gang Yang

**Affiliations:** 1 Department of Radiology, West China Hospital, Sichuan University, 37# Guo Xue Xiang, Chengdu, Sichuan 610041 China; 2 Department of Radiology, Key Laboratory of Birth Defects and Related Diseases of Women and Children of Ministry of Education, West China Second University Hospital, Sichuan University, Chengdu, China; 3 Department of Endocrinology and Metabolism, West China Hospital, Sichuan University, 37# Guo Xue Xiang, Chengdu, Sichuan 610041 China

**Keywords:** Myocardial steatosis, Metabolic syndrome, Subclinical myocardial dysfunction, ^1^H-magnetic resonance spectroscopy

## Abstract

**Background:**

Metabolic syndrome (MetS) is a cluster of metabolic abnormalities that collectively cause an increased risk of type 2 diabetes mellitus (T2DM) and nonatherosclerotic cardiovascular disease. This study aimed to evaluate the role of myocardial steatosis in T2DM patients with or without MetS, as well as the relationship between subclinical left ventricular (LV) myocardial dysfunction and myocardial steatosis.

**Methods and materials:**

We recruited 53 T2DM patients and 20 healthy controls underwent cardiac magnetic resonance examination. All T2DM patients were subdivide into two group: MetS group and non-MetS. LV deformation, perfusion parameters and myocardial triglyceride (TG) content were measured and compared among these three groups. Pearson’s and Spearman analysis were performed to investigate the correlation between LV cardiac parameters and myocardial steatosis. The receiver operating characteristic curve (ROC) was performed to illustrate the relationship between myocardial steatosis and LV subclinical myocardial dysfunction.

**Results:**

An increase in myocardial TG content was found in the MetS group compared with that in the other groups (MetS vs. non-MetS: 1.54 ± 0.63% vs. 1.16 ± 0.45%; MetS vs. normal: 1.54 ± 0.63% vs. 0.61 ± 0.22%; all p < 0.001). Furthermore, reduced LV deformation [reduced longitudinal and radial peak strain (PS); all p < 0.017] and microvascular dysfunction [increased time to maximum signal intensity (TTM) and reduced Upslope; all p < 0.017)] was found in the MetS group. Myocardial TG content was positively associated with MetS (r = 0.314, p < 0.001), and it was independently associated with TTM (β = 0.441, p < 0.001) and LV longitudinal PS (β = 0.323, p = 0.021). ROC analysis exhibited that myocardial TG content might predict the risk of decreased LV longitudinal myocardial deformation (AUC = 0.74) and perfusion function (AUC = 0.71).

**Conclusion:**

Myocardial TG content increased in T2DM patients with concurrent MetS. Myocardial steatosis was positively associated with decreased myocardial deformation and perfusion dysfunction, which may be an indicator for predicting diabetic cardiomyopathy.

## Introduction

Current literature outlines that the excessive accumulation of lipid in cardiomyocytes (myocardial steatosis) is bound to facilitate myocardial lipotoxic injury, which, plays an important role in the development of diabetic cardiomyopathy [1–3]. On the other hand, metabolic syndrome (MetS) is a cluster of risk factors such as central obesity, hyperglycemia, dyslipidemia and hypertension that collectively increase the risk of type 2 diabetes mellitus (T2DM) and cardiovascular disease [4]. Central obesity is one of the most evident clinical features of MetS. Therefore, its development has a prominent role in MetS diagnosis [5]. Chronic inflammation caused by central obesity has been described as an essential factor in the occurrence and development of MetS, and the transition of MetS to cardiovascular disease [6]. Moreover, ectopic fat accumulates around the viscera and regularly enters tissues with only minor amount of adipose tissue such as the heart [7]. At present, there are few studies investigating the myocardial steatosis in T2DM patients with concurrent MetS, and its influence on subclinical cardiac dysfunction.

In recent decades, cardiac magnetic resonance (CMR) imaging has been commonly used in clinical practice, which can provide various characteristics of cardiac structure and myocardial tissue [8–13]. More specifically, feature tracking and first-pass perfusion of CMR imaging have been used to measure myocardial deformation and to detect microvascular dysfunction. On the other hand, proton Magnetic Resonance Spectroscopy (^1^H-MRS) can quantitatively detect triglyceride (TG) content in the myocardium. Therefore, this study aimed to evaluate myocardial steatosis using CMR in T2DM patients with or without concurrent MetS and to investigate the association between left ventricular (LV) subclinical myocardial dysfunction and myocardial steatosis.

## Methods and materials

### Study population

Initially, we prospectively enrolled 92 patients, who were diagnosed with T2DM according to the World Health Organization standards, between June 2017 and May 2019 [14]. Exclusion criteria were as follow: [1] contraindication of CMR; [2] known cardiovascular disease or congenital heart disease; [3] presence of dyspnea, chest pain, palpitation or other cardiovascular disease-related symptoms; and [4] impaired hepatic function or a history of liver disease. Following these criteria, a total of 53 T2DM patients (31 males and 22 females; mean age 54.49 ± 11.16 years) were finally included in this study. In addition, age-, sex-, and body mass index-matched healthy volunteers were recruited in the controls group. Exclusion criteria for the control group were as follows: [1] DM or impaired glucose tolerance; [2] known acute or chronic disease such as hypertension; [3] disease-hyperlipidemia; [4] electrocardiogram abnormalities; and [5] cardiovascular abnormalities detected by CMR (perfusion defect, local, or diffuse myocardial late-gadolinium enhancement, abnormal ventricular motion, valvular stenosis, etc.). Hence, 20 healthy controls (11 males and 9 females; mean age 50.95 ± 10.185 years) were included in this study. Consequently, all T2DM patients and controls underwent CMR provided that they have provided their informed written consent. The study protocol was approved by the West-China hospital of Sichuan University Biomedical Research Ethics Committee.

Clinical characteristics, medication, and serum biochemical indexes of all patients and healthy controls were collected. Blood pressure was measured approximately 20 min before CMR examinations when the subject was in a relaxed state. Blood sampling for serum biochemical indexes was performed within 1 week of the CMR scan without changing the subject’s medication regimen.

Adhering to the definition of MetS by the International Diabetes Federation (2005), we divided T2DM patients into MetS and non-MetS groups [15]. In this definition, central obesity is considered an essential diagnostic element for MetS, and be defined as waist circumference of ≥ 90 cm for males and ≥ 80 cm for females. In addition, the presence of any two of these factors is sufficient for the diagnosis of MetS: (a) increased plasma TG levels (> 150 mg/dL [1.7 mmol/L]) or specific treatment for this lipid abnormality; (b) reduced high-density lipoprotein (HDL)-cholesterol (< 40 mg/dL [1.0 mmol/L] in males; < 50 mg/dL [1.3 mmol/L] in females) or specific treatment for this lipid abnormality; (c) increased blood pressure (systolic ≥ 130 mm Hg and/or diastolic ≥ 85 mm Hg) or treatment of previously diagnosed hypertension; and (d) increased fasting plasma glucose levels (> 100 mg/dL [5.6 mmol/L]) or previously diagnosed T2DM.

### CMR scanning protocol

All subjects were examined using a 3.0-T whole-body scanner (Skrya; Siemens Medical Solutions, Erlangen, Germany) in the supine position. A dedicated two-element cardiac-phased array coil was used for signal detection. Furthermore, a standard ECG-triggering device was used and end-inspiratory breath holding were performed. Following a survey scan, cine images such as long-axis four-chamber views and short-axis two-chamber views were acquired using a steady-state free-precession sequence (TR/TE 39.34/1.22 ms, flip angle 38°, slice thickness 8 mm, field of view 360 × 300 mm^2^, matrix size 256 × 166). Regarding first-pass perfusion imaging, gadobenate dimeglumine (MultiHance; Bracco, Milan, Italy) was intravenously injected at a dose of 0.2 ml/kg body weight at an injection rate of 2.5–3.0 mL/s, followed by a 20 ml saline flush at a rate of 3.0 ml/s. Consequently, first-pass perfusion images were acquired using an inversion-recovery echo-planar imaging sequence (TR/TE 163.00/0.98 ms, flip angle 10°, slice thickness 8 mm, field of view 360 × 270 mm^2^, matrix size 256 × 192) with three standard short-axis slices (apical, middle, and basal), as well as basal slices do not cover the mitral valve level.

**Fig. 1:**
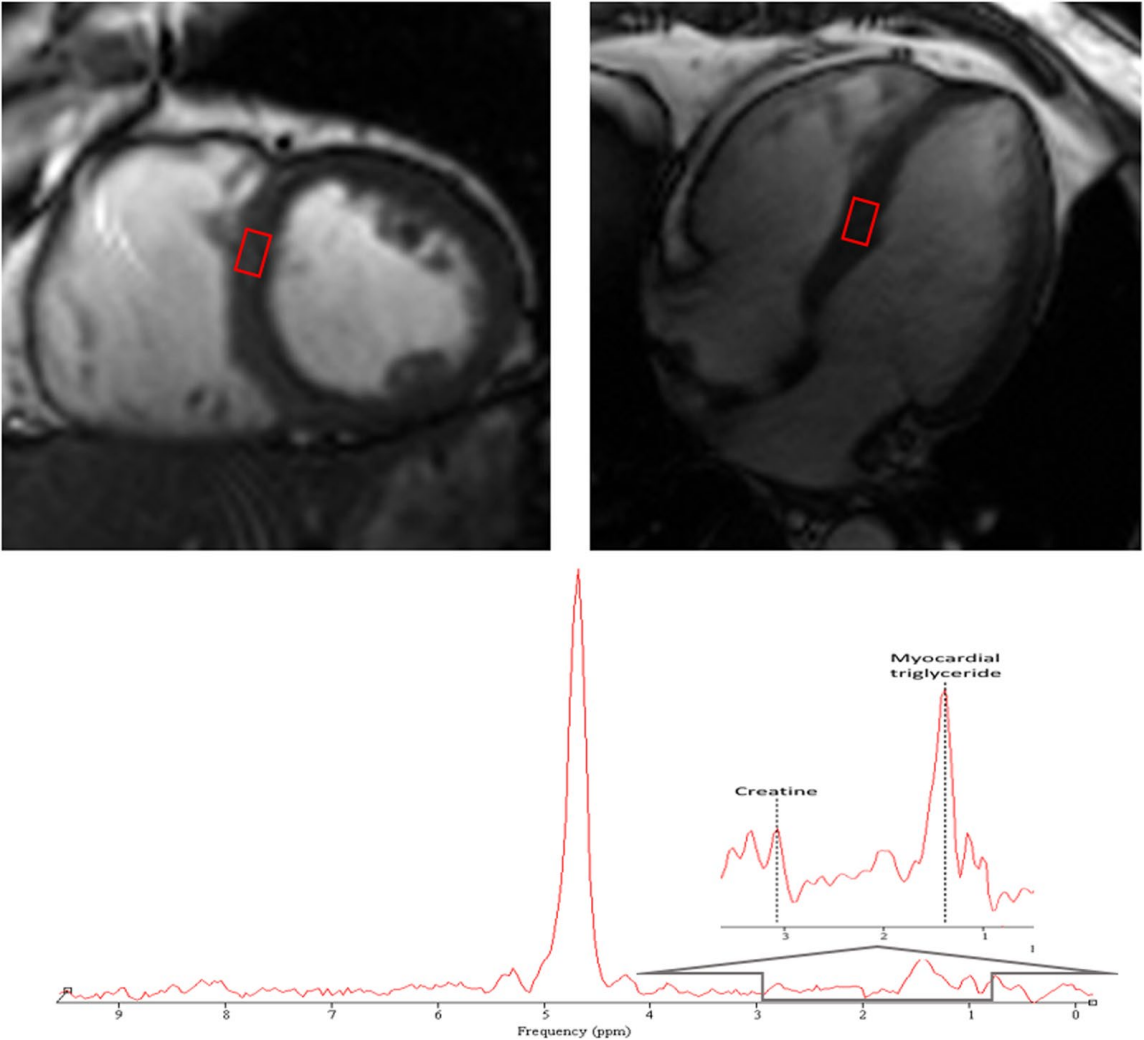
Measurement of myocardial triglyceride content by 1H-MRS. Left 4-chamber and 2-chamber cardiac image. The signal voxel was positioned at the interventricular septum in meddle slice. Myocardial triglyceride content was calculated as a percentage related to water and expressed

^1^H-MRS were performed to obtain the myocardial TG content using a standard flex-coil for signal reception. Voxel positioning was performed in the standard 4-chamber view and 2-chamber short-view, and a single voxel was positioned on the interventricular septum in the meddle slice (Fig. 1). Spectroscopic data were acquired with ECG triggering and respiratory navigator echoes to minimize motion artifacts. We performed two scans using the abovementioned sequence. During the first scan, the water suppression mode was used to eliminate the imbibition caused by water from the signal of interest. During the second scan, the water suppression mode was not used, a water signal is obtained. Spectral data collection was performed with the PRESS sequence (TR/TE 560/33 ms, average 4). All 1H-MRS data were analyzed using a Java-based software (jMRUI, version 6.0, Leuven, Belgium). TG content was calculated as a percentage related to water and expressed as following:

$${{\left( {\text{signal amplitude of TG}} \right)} \mathord{\left/ {\vphantom {{\left( {\text{signal amplitude of TG}} \right)} {\left( {\text{signal amplitude of water}} \right)}}} \right. \kern-0pt} {\left( {\text{signal amplitude of water}} \right)}}\, \times \, 100$$

### CMR data analysis

We uploaded all acquired images data to an offline workstation using a semi-automated software (Cvi42; Circle Cardiovascular Imaging, Inc., Calgary, Canada). Endocardial and epicardial traces were delineated manually by two experienced radiologists in the serial short-axis slices during the end-diastolic and end-systolic phases. LV functional parameters and LV mass were automatically determined. LV remodeling was characterized by the ratio of LV mass to LVEDV (LVMVR). The LV global function index (LVGFI) was calculated using the following formula:

$$ \begin{aligned}{\text{LVGFI}}\, &= \,\{ {\text{LVSV}}/([{\text{LVEDV}}\, + \,{\text{LVESV}})/ 2\, \\ &\quad+ \,\left( {{\text{LV mass}}/ 1.0 5} \right)]\} \, \times \, 100\end{aligned} $$

To evaluate LV microvascular perfusion, blood pools as well as endocardial and epicardial traces of the meddle slice of first-pass perfusion images were delineated manually (in order to match the voxel level of ^1^H-MRS), and a region of interest was placed over the blood pool as a means of contrast. In addition to myocardial and blood pooled time-signal intensity curves, semi-quantitative perfusion parameters were obtained such as upslope, maximum signal intensity (MaxSI), and time to maximum signal intensity (TTM).

### Variability analysis

To determine intra-observer variability, LV deformation and perfusion parameters in 30 random cases that included 20 T2DM patients and 10 normal controls were measured twice in 1-week intervals by a radiologist. Then, a second investigator, who was blinded to the first investigator’s results, reanalyzed the measurements. Finally, the interobserver variability was assessed on the basis of the two investigators’ results. The two radiologists were blinded to the status (DM vs control, DM with MetS vs. DM without MetS).

### Statistical analysis

Statistical analyses were performed with commercially available SPSS (version 21.0 for windows; SPSS, Inc., Chicago, IL, USA). Results are expressed as the mean ± standard deviation. One-way analysis of variance test was performed to evaluate the differences among the following groups: T2DM with MetS, T2DM without MetS and control. Based on Bonferroni’s correction for multigroup comparisons, p-values of < 0.017 were considered as statistically significant. Spearman’s and Pearson’s correlation analysis were conducted to identify the relationship between myocardial steatosis and cardiac deformation. Moreover, multivariable stepwise linear regression analysis was employed to identify the relationship between myocardial TG content and subclinical cardiac dysfunction. Receiver operating characteristic curve (ROC) analysis was conduncted to predict myocardial steatosis to LV subclinical myocardial dysfunction.

## Results

### Patient characteristics and metabolic parameters

Of the 53 T2DM patients, 23 were included in the non-MetS (15 males, mean age 54.85 ± 10.87 years) and 30 in the MetS group (16 males, mean age 54.48 ± 9.61 years). Table 1 presents their baseline characteristics, metabolic parameters, and medication. Weight and BMI were found to be higher in the MetS group than in the non- MetS group and the control group, whereas systolic blood pressure was higher in the MetS group than in the control group.

**Table 1:** Baseline and metabolic parameters T2DM patients with or without metabolic syndrome and the normal controls

	Normal (n = 20)	Non-MetS (n = 23)	MetS (n = 30)
Baseline characteristics			
Age (y)	50.95 ± 10.185	54.85 ± 10.87	54.48 ± 9.61
Male (n)	10(50.0%)	15(65.2%)	16(53.3%)
High (cm)	162.70 ± 7.08	162.85 ± 9.23	164.13 ± 7.29
Weigh (kg)	61.30 ± 5.96	58.52 ± 8.22	66.54 ± 8.55*β
BMI	23.14 ± 1.49	22.03 ± 2.30	24.71 ± 3.05β
Systolic blood pressure (mmHg)	119.45 ± 7.22	130.36 ± 16.86	132. ± 19.22*
Diastolic blood pressure (mmHg)	78.75 ± 7.81	76.42 ± 7.67	80.09 ± 10.53
Duration of diabetes (y)	–	7.32 ± 7.24	7.90 ± 4.59
Waist circumference (cm)	–	83.33 ± 5.79	93.37 ± 1.05
Metabolic characteristics			
HbA1c (%)	5.38 ± 0.34	6.47 ± 2.87*	6.75 ± 2.76*
GLU (mmol/l)	5.07 ± 0.43	7.11 ± 5.12*	5.70 ± 3.94
TG (mmol/l)	1.03 ± 0.27	1.14 ± 0.41	1.76 ± 1.64*
TC (mmol/l)	4.30 ± 0.57	4.29 ± 0.86	4.59 ± 0.83
HDL (mmol/l)	1.31 ± 0.28	1.47 ± 0.34	1.23 ± 0.36
LDL (mmol/l)	2.71 ± 0.53	2.36 ± 0.73	2.70 ± 0.74
Creatinine (μmol/l)	–	66.20 ± 12.59	73.97 ± 22.15
GFR (30 ml/min)	–	99.84 ± 10.32	95.85 ± 19.17
AST (U/l)	–	23.00 ± 12.07	25.58 ± 15.82
ALT (U/l)	–	24.53 ± 8.94	23.04 ± 8.94
Uric acid (μmol/l)	340.00 ± 37.04	331.81 ± 60.49	359.88 ± 95.95
Medication, n (%)			
Insulin	–	7 (33%)	10 (31%)
Metformin	–	11 (52%)	19 (59%)
Sulfonylurea	–	2 (9%)	9 (28%)
α-Glucosidase inhibitor	–	5 (24%)	16 (50%)
ACEI	–	0	4 (13%)
Statin	–	2 (9%)	7 (22%)

The values are the mean ± SD. *BMI* body mass index, *HbA1c* glycated hemoglobin, *Glu* glucose, *TG* triglycerides, *TC* triglyceride, *HDL* highdensity lipoprotein cholesterol, *LDL* low-density lipoprotein cholestero, *GFR* glomerular filtration rate, *AST* aspartate transaminase, *ALT* alanine transaminase

*P < 0.017 versus normal group

β*P* < 0.017 versus T2DM in non-MetS group

HbA1c was higher in T2DM patients than in normal controls, and serum TG content was higher in the MetS group than in the control group (1.76 ± 1.64 mmol/L vs. 1.03 ± 0.27 mmol/l; p < 0.001). In terms of medication, the MetS group were more likely to received treatment for this lipid abnormality. The remaining baseline and metabolic characteristics showed no statistically significant difference among all the three groups.

### CMR ^1^H-MRS analysis

**Fig. 2:**
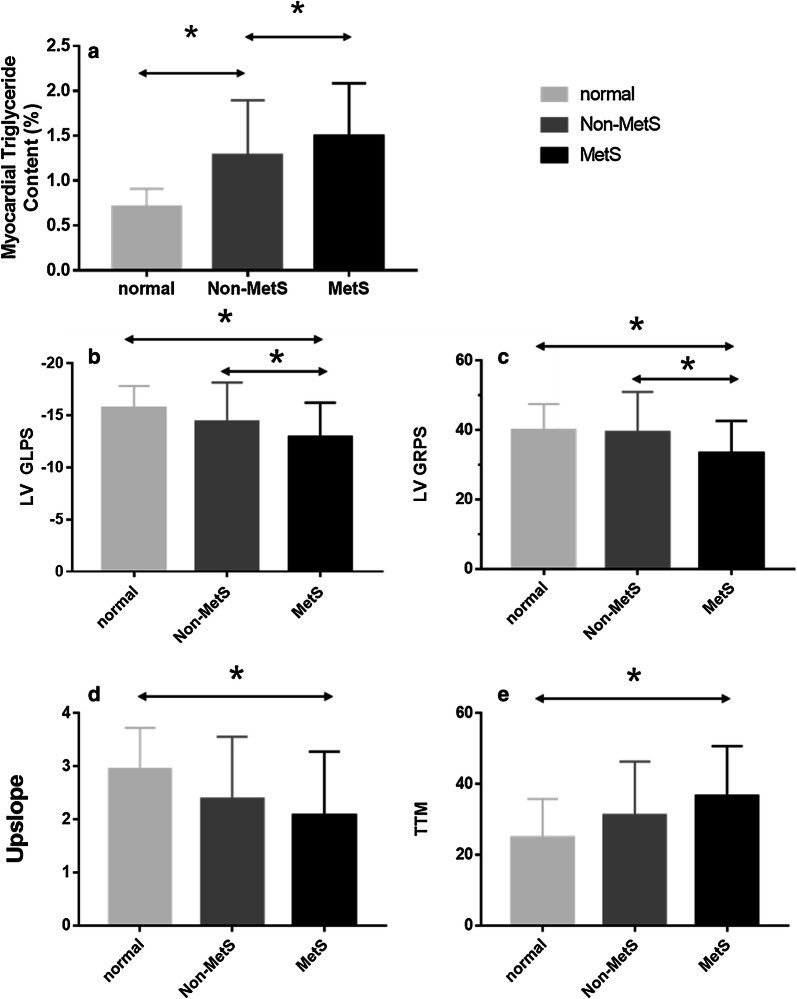
Differences in myocardial triglyceride content (**a**), LV longitudinal PS (**b**), LV radial PS (**c**), upslope (**d**) and TTM (**e**) among patients in T2DM with MetS, T2DM without MetS, and normal subjects. *p < 0.017

The result of the myocardial TG content are summarized in Table 2. The MetS group had significantly higher myocardial TG content than that of the non-MetS group (1.54 ± 0.63% vs. 1.16 ± 0.45%, p < 0.001) and the control (1.54 ± 0.63% vs. 0.61 ± 0.22%, p < 0.001; Fig. 2a). Furthermore, the non-MetS group had a significantly higher myocardial TG content than the control group (1.16 ± 0.45% vs. 0.61 ± 0.22%, p < 0.001).

**Table 2:** CMR parameters for T2DM patients with or without metabolic syndrome and the normal controls

	Normal (n = 30)	Non-MetS (n = 23)	MetS (n = 30)
^1^H-MRS			
Myocardium TG (%)	0.61 ± 0.22	1.16 ± 0.45*	1.54 ± 0.63*β
Cardiac function			
LVEDV (ml/m^2^)	75.89 ± 12.94	69.92 ± 14.33	66.20 ± 12.10*
LVESV (ml/m^2^)	28.57 ± 6.17	25.17 ± 7.24	25.98 ± 6.54
LVSV (ml/m^2^)	47.30 ± 8.12	44.74 ± 8.95	40.22 ± 8.945*
LVEF (%)	62.47 ± 4.09	64.32 ± 6.06	60.82 ± 7.62
LV mass (g/m^2^)	75.93 ± 14.33	82.91 ± 26.11	92.25 ± 22.81*
LVGFI (%)	51.97 ± 6.60	47.33 ± 6.75	46.02 ± 9.43*
LVMVR	0.55 ± 0.13	0.65 ± 0.15	0.76 ± 0.22*
LV stain			
Radial PS (%)	39.85 ± 7.64	39.98 ± 12.05	33.28 ± 9.00*β
Circumferential PS (%)	− 20.41 ± 2.50	− 19.34 ± 3.48	− 19.38 ± 2.32
Longitudinal PS (%)	− 15.71 ± 2.10	− 14.78 ± 3.48	− 12.67 ± 3.46*β
First perfusion			
Upslope	2.93 ± 0.78	2.45 ± 1.20	2.10 ± 1.19*
TTM (s)	24.77 ± 11.01	30.44 ± 14.71	36.09 ± 14.57*
MaxSI	24.19 ± 6.14	23.46 ± 9.13	20.65 ± 7.94

The values are the mean ± SD. *LVEDV* left ventricular end-diastolic volume, *LVESV* left ventricular end-systolic volume, *LVSV* left ventricular stroke volume, *LVEF* left ventricular ejection fraction, *LVGFI* left ventricular global function index, *LVMVR* LV mass to LV end diastolic volume ratio, *PS* peak strain, *TTM* time to maximum signal intensity

*P < 0.017 versus normal group

β*P* < 0.017 versus T2DM in non-MetS group

### CMR imaging analysis

Regarding LV function and deformation, LVEDV, LVESV and LVGFI (all p < 0.001) were lower in the MetS group compared to the control group, whereas LV mass (92.25 ± 22.81 g/m^2^ vs. 75.93 ± 14.33 g/m^2,^p < 0.001) and LVMVR (0.76 ± 0.22 vs. 0.55 ± 0.13, p < 0.001) were higher in the MetS group than in the control group.

The global longitudinal peak strain (PS) (MetS vs. non-MetS: − 12.67 ± 3.46% vs. − 14.78 ± 3.48%; MetS vs. control: − 12.67 ± 3.46% vs. − 15.71 ± 2.10%, all p < 0.001) (Fig. 2b) and global radial PS (MetS vs. non-MetS: 33.28 ± 9.00% vs. 39.98 ± 12.05%; MetS vs. normal: 33.28 ± 9.00% vs. 39.85 ± 7.64%, all p < 0.001) (Fig. 2c) were lower in the MetS group than in the non-MetS and control groups. There was no statistically significant difference in myocardial deformation between the non-MetS and control group.

T2DM patients in MetS group had a significantly lower perfusion upslope (2.10 ± 1.19 vs. 2.93 ± 0.78, p < 0.001) (Fig. 2d) but higher TTM values (36.09 ± 14.57 s vs. 24.77 ± 11.01 s, p < 0.001) (Fig. 2e) than the control group. However, no difference was observed in these values compared with the non-MetS group. In fact, there was no significant difference in terms of all perfusion parameters between the non-MetS and the control group.

### Association between MetS, myocardial steatosis, and myocardial function

**Fig. 3:**
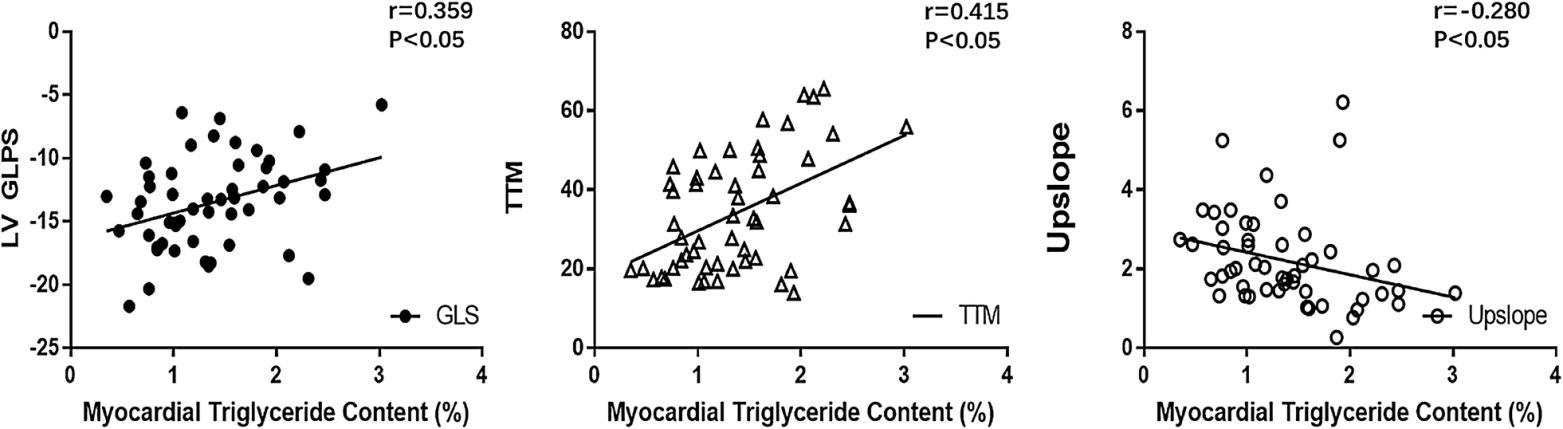
Relationship between myocardial triglyceride content and LV longitudinal PS, TTM and upslope

Spearman correlation analysis showed that MetS had a positive correlation with myocardial TG content (r = 0.314, p < 0.05). Furthermore, myocardial TG content was positively associated with LV longitudinal PS (r = 0.359, p < 0.05), TTM (r = 0.415, p < 0.05), and negatively associated with upslope (r = − 0.280, p < 0.05) (Fig. 3). There was no significant correlation between MetS and other cardiac-related parameters (all p > 0.05).

Multivariable stepwise linear regression analysis indicated that myocardial TG content (β = 0.441, p < 0.001) and diastolic blood pressure (β = 0.254, p = 0.041) were independently associated with the TTM (Model.3: R^2^ = 0.459), and the myocardial TG content (β = 0.323, p = 0.021) was also independently associated with LV longitudinal PS (Model.3: R^2^ = 0.323) (Table 3).

**Table 3:** Multivariable associations between cardiac parameters and myocardial triglyceride content

	TTM				Longitudinal PS			
	Beta	p value	R^2^	p value	Beta	p value	R^2^	p value
Model 1								
Myocardial TG content	0.415	0.020	–	–	0.339			
Model 2								
Myocardial TG content	0.415	< 0.001	0.413	< 0.001	0.308	0.025	0.308	0.025
Duration of diabetes	–	–			–	–		
BMI	–	–			–	–		
Model 3								
Myocardial TG content	0.441	< 0.001	0.459	< 0.001	0.323	0.021	0.323	0.021
Duration of diabetes	–				–	–		
BMI	–				–	–		
Systolic blood pressure	–				–	–		
Diastolic blood pressure	0.254	0.041			–	–		
Age	–				–	–		
Glu					–	–		

**Fig. 4:**
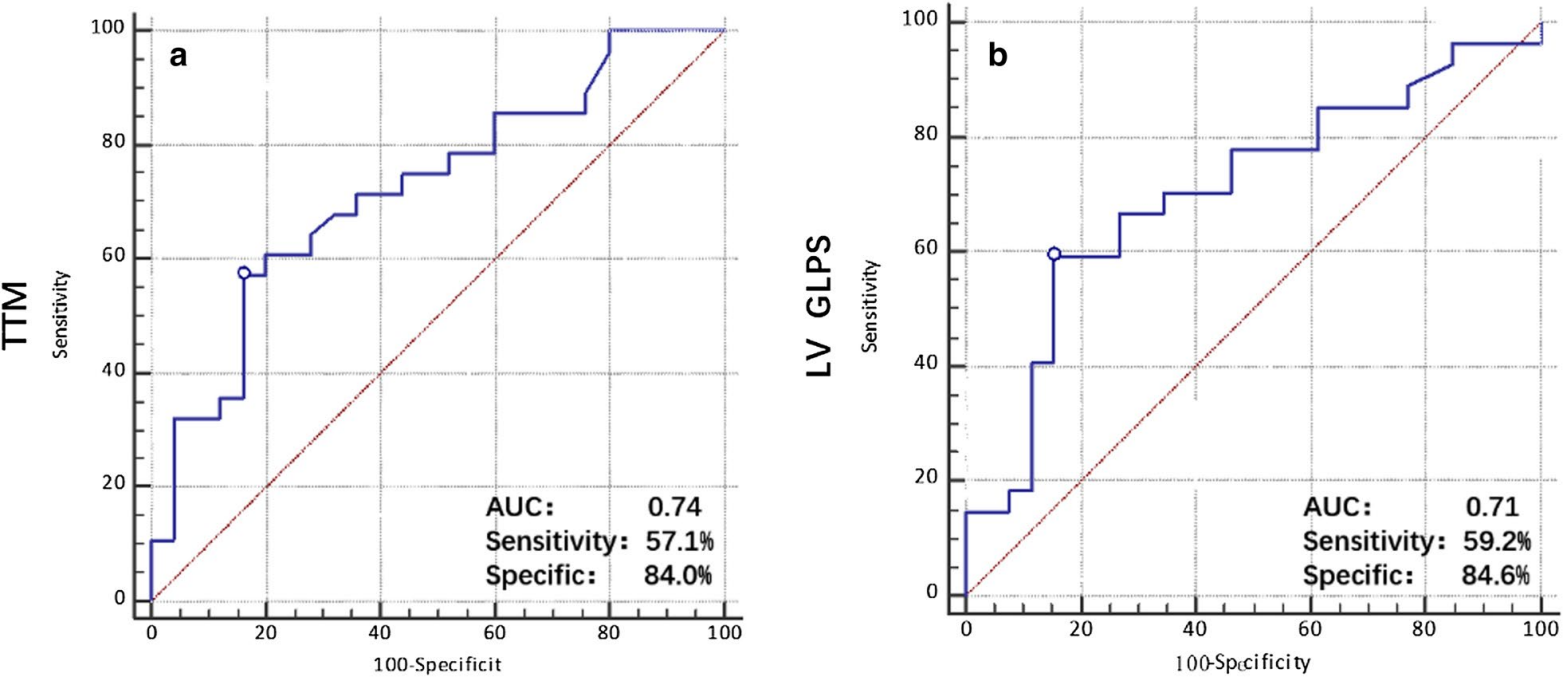
Receiver operating characteristic curve (ROC) analysis to predict the relationship between the myocardial triglyceride content and TTM (**a**), LV longitudinal PS (**b**)

ROC analysis demonstrated that the cutoff value for myocardial TG content that predicted the risk of myocardial microvascular perfusion dysfunction (sensitivity = 57.1%, specificity = 84.0%, and AUC = 0.74) (Fig. 4a) and longitudinal myocardial deformation (sensitivity = 59.2%, specificity = 84.6%, and AUC = 0.71) (Fig. 4b) was 1.56.

### Inter- and intra-observer variability

Table 4 summarizes the inter- and intra-observer variability for LV deformation and first-pass perfusion analysis. The ICCs for intra- and interobserver variability were 0.923–0.959 and 0.883–0.955, respectively, in LV deformation and 0.977–0.991 and 0.982–0.993 respectively, in first-pass perfusion, suggesting that both techniques are in agreement.

**Table 4:** Inter- and intra-observer variability of first-perfusion and tissue tracking

	Intra-observer (n = 30)	95% CI	Inter-observer (n = 30)	95% CI
Radial PS (%)	0.959	0.901–0.982	0.955	0.905–0.979
Circumferential PS (%)	0.932	0.851–0.969	0.938	0.866–0.972
Longitudinal PS (%)	0.923	0.831–0.965	0.883	0.745–0.947
Upslope	0.995	0.990–0.998	0.993	0.982–0.997
TTM (s)	0.991	0.980–0.996	0.985	0.967–0.993
MaxSI	0.977	0.944–0.995	0.982	0.951–0.993

## Discussion

In this study, the following principal findings were obtained: (1) T2DM patients with MetS may be more likely to present myocardial steatosis; (2) there was a decreased of LV deformation and microcirculation perfusion in T2DM patients with MetS; and (3) an increased myocardial TG content was associated with the reduce of LV longitudinal deformation and microvascular perfusion, and it might be an appropriate predictor of the myocardium damages.

As a noninvasive technique, ^1^H-MRS MRS can investigate the cardiac metabolism in vivo, thereby quantitatively detecting metabolites, including fatty acids (FA), creatine etc. Therefore, ^1^H-MRS can help diagnose myocardial steatosis at an early stage and facilitate the targeted treatment of diabetes mellitus.

The pathological mechanism of diabetic cardiomyopathy is complex and multifactorial. Recent studies have indicated that myocardial lipotoxic injury as a result of lipid oversupply plays an important role in diabetic heart disease [16, 17]. In this study, we identified the progression of myocardial steatosis (increasing of myocardial TG content) in T2DM patients, particularly in those with concurrent MetS, and it was positively associated with MetS. We suspect that this was due to insulin resistance, central obesity, and increased serum FA content, which leads to increased myocardial FA delivery and uptake in T2DM patients [18]. Furthermore, central obesity was one of the most critical factors that facilitated excessive myocardium lipids deposition in MetS patients [19]. Therefore, T2DM patients with concurrent MetS are more prone to developing myocardial steatosis.

In addition, we observed that only the MetS group exhibited reduced LV longitudinal and radial PS, which might mean reduction of early myocardial diastolic function. According to the distribution of myocardial fibers, the longitudinal myocardial fibers are predominantly located in the sub-endocardium and are most susceptible to early microvascular ischemia [20]. As the central clinical features of MetS, insulin resistance and central obesity increase the inflammation and oxidative stress, thereby inducing endothelial dysfunction and cardiomyocyte apoptosis reducing the ability of myocardial deformation, and ultimately damaging the myocardium; this results in decreased LV deformation of varying degrees [21]. Moreover, our observations related to upslope and TTM indicated that microcirculation function considerably decreased in T2DM patients with MetS. Whereas, despite the reduction trend presented, there was no statistical difference in the non-MetS group and normal control group. It means that when T2DM patients are accompanied by MetS, their myocardial microvascular is reduced, we can presume that compared with subcutaneous fat, central obesity may cause more serious myocardiual damage because it is associated with the adverse remodeling of intramural coronary arterioles. Therefore, the impaired vasodilation further reduced myocardial microvascular perfusion8 [22–28].

An additional finding in our study was that the T2DM in MetS group exhibited a tendency of concentric LV remodeling and reduced of LVGFI. In contrast, the T2DM patients in non-MetS group did not present similar myocardial structural changes. Concentric LV remodeling is considered to be an early sign of obesity-related cardiac remodeling before LV hypertrophy occurs [29]. It has been reported that LV wall thickening is associated with radial strain [30]. Therefore, LV concentric remodeling can lead to myocardial hypertrophy, and radial strain can be reduced to a varying degree. In our study, the LV global radial PS was decreased in the MetS group. In addition, we hypothesize that in addition to insulin resistance and central obesity, other pathological disorders secondary to metabolic ones, such as hypertension, hyperlipidemia, and hyperglycemia, may continue to cause more serious myocardial lesions in T2DM patients with concurrent MetS than in those without MetS [31].

A pervious study has identified myocardial steatosis may play an important mechanistic role in the development of diastolic dysfunction in women with microvascular dysfunction and no obstructive CAD [32]. In our study we found the similar mechanism in T2DM patients. Our results show the association between myocardial steatosis and longitudinal PS, this also confirms that T2DM patients are prone to early diastolic dysfunction [8]. Moreover, using electrocardiographically gated gradient-echo sequence with velocity encoding, Rijzewijk et al. found that myocardial steatosis is an independent predictor of early diastolic dysfunction in uncomplicated T2DM [33]. Our present study also reached a similar conclusion using CMR, in that the correlation between myocardial TG content and myocardial deformation decreased in T2DM patients with MetS. Besides myocardial deformation, we also identified that an increase in myocardial TG content is negatively related to myocardial microvascular perfusion function, regardless of patients’ age, BMI, heart rate, duration of diabetes, plasma glucose, and blood pressure, and myocardial TG content had a moderately predictive effect on the myocardial microvascular perfusion. Furthermore, Nyman et al. found that MetS was associated with LV diastolic dysfunction, and our research indicated that when T2DM is accompanied with MetS, the injury of LV deformation and microvascular perfusion is aggravated ([7]). To order to adapt to metabolic disorder, the myocardium maintains a high oxygen consumption rate and FA oxidation rate under conditions of insulin resistance, visceral adiposity, and increased serum dietary FA content, thus facilitating the accumulation of intracellular TG in the myocyte cytoplasm [34, 35]. Intracellular TG is relatively inert, but an increase in its content reflects a respective increase in anaerobic oxidation of FA and accumulation of lipotoxic intermediates such as ceramide and diacyl-glycerol [18, 36–38]. These lipotoxic intermediates have been shown to activate signaling pathways that affect ATP production, insulin sensitivity, and apoptosis, but they also trigger replacement fibrosis and myocardial contractile dysfunction [18, 39]. Therefore, we believe that T2DM patients with concurrent MetS are more prone to developing myocardial lipotoxic injury, thus suggesting that T2DM and MetS have synergistic effects on myocardial degeneration and myocardial injury.

## Limitations

There are several limitations to our study. First of all, this study was a single center study; hence, an assemble bias may have influenced the acquired results. Second, because we did not perform secondary CMR examinations or other follow-up investigations, our results need to be verified by longitudinal studies on T2DM patients. Hence, it is our principle focus to verify these findings in future follow-up studies.

## Conclusion

Our study found that even when the cardiac function of patients with T2DM is preserved, the combined MetS may increase the reduction of myocardial deformation and myocardial perfusion, and these changes in myocardial structure are related to the degree of myocardial steatosis. Meanwhile, myocardial triglyceride content might be a useful indicator to predicting diabetic cardiomyopathy. Therefore, it is suggested that we should pay more attention to myocardial steatosis in clinically diabetic patients with metabolic syndrome, and reducing myocardial steatosis may also help prevent the progression of diabetic cardiomyopathy.

## Data Availability

The datasets used and analyzed during the current study are available from the corresponding author on reasonable request.

## Abbreviations

T2DM: Type 2 diabetes mellitus
MetS: Metabolic syndrome
BMI: Body mass index
HDL: High density lipoprotein
TG: Plasma triglycerides
CMR: Cardiovascular magnetic resonance
LV: Left ventricular
EDV: End-diastolic volume
ESV: End-systolic volume
SV: Stroke volume
EF: Ejection fraction
TTM: Time to maximum signal intensity
MaxSI: Max signal intensity
^1^H-MRS: Proton Magnetic Resonance Spectroscopy
PS: Peak strain
ROC: The receiver operating characteristic curve
